# Pipetting-based immunoassay for point-of-care testing: Application for detection of the influenza A virus

**DOI:** 10.1038/s41598-019-53083-8

**Published:** 2019-11-13

**Authors:** Ji Yeong Noh, Sun-Woo Yoon, Youngji Kim, Thi Van Lo, Min-Ju Ahn, Min-Chul Jung, Tran Bac Le, Woonsung Na, Daesub Song, Van Phan Le, Seungjoo Haam, Dae Gwin Jeong, Hye Kwon Kim

**Affiliations:** 1 Department of Microbiology, College of Natural Sciences, Chungbuk National University, Cheongju, Republic of Korea; 2 Infectious Diseases Research Center, Korea Research Institute of Bioscience and Biotechnology, Daejeon, Republic of Korea; 3 Bio-Analytical Science Division, University of Science and Technology (UST), Daejeon, Republic of Korea; 4 College of Veterinary Medicine, Chungbuk National University, Cheongju, Republic of Korea; 5 Department of Pharmacy, College of Pharmacy, Korea University, Sejong, Republic of Korea; 6 Department of Microbiology and Infectious Diseases, College of Veterinary Medicine, Vietnam National University of Agriculture, Hanoi, Vietnam; 7 Department of Chemical and Biomolecular Engineering, Yonsei University, Yonsei-ro 50, Seoul, 03722 Republic of Korea; 8 College of Veterinary Medicine, Chonnam National University, Gwangju, Korea

**Keywords:** Assay systems, Infectious-disease diagnostics

## Abstract

Point-of-care tests (POCT) for pathogens are considered important for low-resource countries and facilities. Although lateral flow immunoassays (LFIA) have many advantages including speed and ease of use, their sensitivity is limited without specific equipment. Furthermore, their response cannot be enhanced through enzymatic reactions. Owing to these limitations, LFIAs have not yet been generally adopted as the standard protocol for *in vitro* analysis of infectious pathogens. We aimed to develop a novel pipetting-based immunoassay using a removable magnetic ring-coupled pipette tip. The “magnetic bead-capture antibody-targeted protein complex” was simply purified by pipetting and quantified by enzymatic colour development or using a lateral flow system. This pipetting-based immunoassay was applied to detect the nucleoprotein (NP) of the influenza A virus. Using an HRP-conjugated monoclonal antibody as a probe, the assay allowed for specific and sensitive detection. Furthermore, when this assay was applied exclusively for antigen capture in the lateral flow system, the limit of detection improved 100-fold and displayed greater sensitivity than the lateral flow system alone. Therefore, the pipetting-based immunoassay may be potentially used as a sensitive POCT to clinically detect a target antigen.

## Introduction

Point-of-care tests (POCT) for pathogens are considered imperative for low-resource countries and facilities^1^. In addition, initial screening for infectious pathogens is epidemiologically critical to prevent and control disease spread among the population. Therefore, it is essential to develop an easy and convenient POCT that is applicable in various settings including local hospitals, veterinary clinics, and animal farms.

Lateral flow immunoassays (LFIA) have been widely developed and commercialised as the most popular POCTs owing to their ease of use and rapid yield of results^2^. LFIAs use coloured labels such as gold nanoparticles for visualisation. Furthermore, fluorescent and magnetic labels have been developed for LFIAs along with specific detection equipment for sensitive and quantitative detection^3–5^. Although LFIAs have many advantages, including speed and ease of use, their sensitivity is limited without specific equipment and it is not possible to enhance the response through enzymatic reactions^3^. Due to this limitation, LFIAs have not yet been generally adopted as the standard protocol for the standard *in vitro* analysis of infectious pathogens.

Since enzymatic reactions are catalytic, enzyme-based colorimetric immunoassays have been widely used for antigen and antibody detection with reliable sensitivity. Among them, the enzyme-linked immunosorbent assay (ELISA) has been applied in different commercial kits. Moreover, ELISA is considered one of the standard *in vitro* assays to detect several infectious diseases in humans^6,7^. ELISA commonly uses solid-phase techniques with microtiter plates (96 wells) containing a covalently bound antigen or antibody^8^. Although ELISA is one of the most popular immunoassays, it has a few limitations as a POCT, especially in resource-limited settings^9^. In addition, the solid-phase binding system of the 96-well microplate comprises multiple reactions and washing steps and is not suitable to analyse small amounts of clinical samples at local hospitals and veterinary clinics.

Microfluidic systems-based POCT have been developed to increase the availability of POCTs in resource-limited settings and as a reliable standard *in vitro* test. Major microfluidic platforms are based on capillary, pressure-driven, centrifugal, electrokinetic, and acoustic liquid propulsion principles, among which linear actuated devices and centrifugal microfluidics have been considered potential next-generation platforms for POCTs^10^. However, there are still practical barriers to clinical application due to the need for specific equipment and complicated fluidic networks. To overcome these barriers, a new POCT device was recently developed based on the volumetric measurement of oxygen generated through an ELISA reaction, called the multiplexed volumetric bar-chart chip^11,12^. Notwithstanding its reliability and ease of use, the photolithography-based fabrication in devices may serve as a limitation for manufacturing these devices.

**Figure 1:**
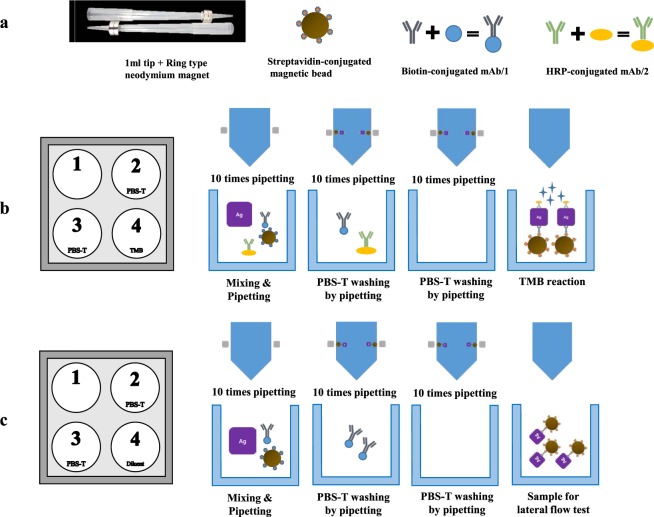
Materials and schematic procedure of the pipetting-based immunoassays. (**a**) Materials for the pipetting-based immunoassays. (**b**) Schematic procedure of the pipetting-based immunoassay for enzymatic colour development. (**c**) Schematic procedure of the pipetting-based immunoassay for antigen capture.

In this study, we developed a novel pipetting-based immunoassay using removable magnetic ring-coupled pipette tip. As shown in Fig. 1, a “magnetic bead-capture antibody-targeted protein complex” was simply purified by pipetting and quantified by an enzyme-based colour reaction and lateral flow test. This simplified protocol is easy to develop and is applicable in low-resource settings. Therefore, its suitability for the POCT was evaluated with the influenza A virus.

## Results

### Equipment optimisation for pipetting-based immunoassay

**Figure 2:**
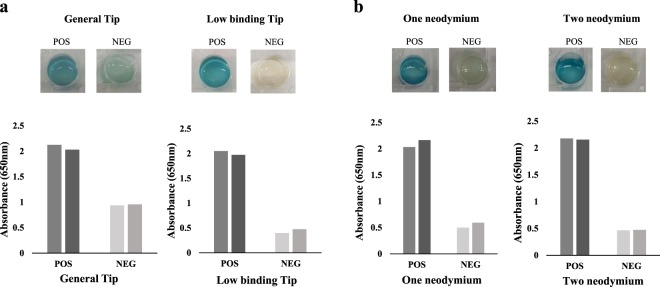
Optimization for Pipetting-based immunoassay. (**a**) Comparison of the non-specific background between general tip and low-binding tip in the pipetting-based immunoassay for enzymatic colour development, POS (positive control) (recombinant NP, 110 µg/mL), NEG, negative control (PBS, pH 7.4). (**b**) Comparison of the non-specific background between one ring-type neodymium magnet and two ring-type neodymium magnets in the pipetting-based immunoassay for enzymatic colour development, POS, positive control (recombinant nucleocapsid protein (NP), 110 µg/mL), NEG, negative control (PBS, pH 7.4).

To select the optimal pipette tip, two types of 1 mL pipette tips were compared for their applicability in the pipetting-based immunoassay targeting nucleoprotein (NP) of influenza A virus: a general tip (OHAUS) and a low-binding tip (Bioneer) were compared using 100 µL of 110 µg/mL recombinant influenza NP protein as the positive control and phosphate buffered saline (PBS) as the negative control. As shown in Fig. 2, non-specific background in the negative control was greater in the general tip group than in the low-binding tip group, yielding average absorbance values of 0.937 and 0.399 in general tip group and low-binding tip group, respectively, at 650 nm. The low-binding pipette tip was superior as it minimised non-specific reactions compared to the general pipette tip. The low-binding pipette tip equipped with two ring-type neodymium magnets also showed a lesser non-specific background, with average absorbance values of 0.399 with two magnets and 0.475 with one magnet, respectively, at 650 nm.

### Limit of detection of the pipetting-based immunoassay for enzymatic colour development

**Figure 3:**
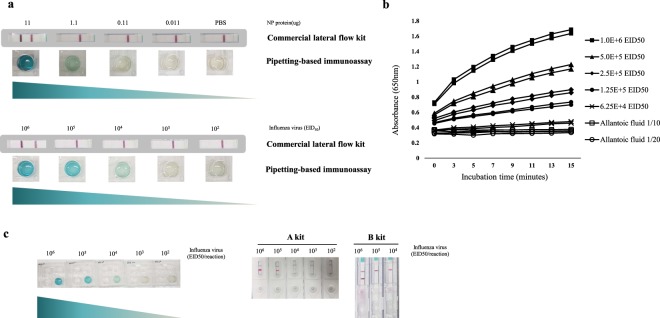
The limit of detection of the pipetting-based immunoassay for enzymatic colour development. (**a**) Limit of detection of the pipetting-based immunoassay for enzymatic colour development using 11 µg, 1.1 µg, 0.11 µg, and 0.011 µg/reaction of the recombinant NP in PBS (pH 7.4), and 10^6^, 10^5^, 10^4^, 10^3^, and 10^2^ EID_50_/reaction of A/swine/Korea/P17-4/2017 isolate in PBS (pH 7.4). (**b**) Time-based colour development measured with two-fold diluted A/swine/Korea/P17-4/2017 isolate (10^7^ EID_50_/mL), 10^6^, 5 × 10^5^, 2.5 × 10^5^, 1.25 × 10^5^, 6.25 × 10^4^ EID_50_/reaction, and negative control as 10 times and 20 times diluted allantoic fluid of specific pathogen-free embryonated eggs with PBS (pH 7.4). The absorbance value of the colour-developed solution was measured at 650 nm in duplicates. (**c**) Comparison of the limits of detection among the pipetting-based immunoassays for enzymatic colour development and two commercial lateral flow kits (kits A and B).

The limit of detection of the pipetting-based immunoassay for enzymatic colour development was measured using the recombinant NP and swine influenza virus (H3N2). The assay could detect up to 1.1 μg/reaction (4.7 ng/μL) of recombinant influenza NP protein and 10^4^ EID_50_/reaction of swine influenza virus (H3N2) (Fig. 3a). Colour development with time was measured using two-fold diluted swine influenza virus (10^7^ EID_50_/ml) and time-dependent colour changes were observed for 15 min (Fig. 3b). On diluting the viral isolates to 6.25 × 10^4^ EID_50_/reaction, enzymatic colour development was distinguishable from allantoic fluid as the negative control. However, the rate of the reaction for the colour development test was lower at that viral concentration. When the limit of detection was compared to that of the commercial lateral flow kits, the pipetting-based immunoassay for enzymatic colour development showed the same limit of detection (10^4^ EID_50_/reaction) with that of the kit A, the value being 100-fold that of the kit B (Fig. 3c).

### Specific reactivity of the pipetting-based immunoassay for enzymatic colour development

**Figure 4:**
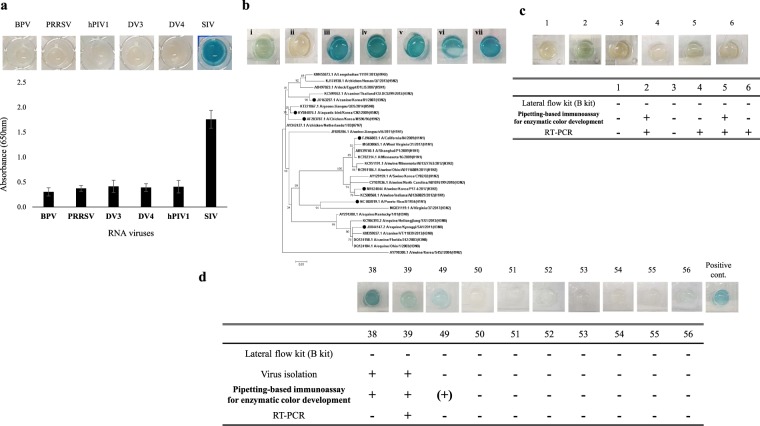
Validation of the pipetting-based immunoassay for enzymatic colour development. (**a**) Specificity test of the pipetting-based immunoassay for enzymatic colour development with other RNA viruses, BPV, PRRSV, hPIV1, DV 3, and 4. Standard deviation is indicated by error bar. (**b**) Detection of various subtype of influenza A viruses by pipetting-based immunoassay for enzymatic colour development: A/canine/Korea/01/2007(i), A/equine/Kyonggi/SA1/2011(ii), A/California/04/2009(iii), A/Puerto Rico/8/1934(iv), A/aquatic bird/Korea/CN2/2009(v), A/Chicken/Korea/MS96/96(vi), and A/swine/Korea/P17-4/2017(vii). The phylogenetic tree was generated by the maximum-likelihood method with 1,000 replicates of bootstrap sampling and the Jones-Taylor-Thornton (JTT) model using MEGA 6^21^. The various subtypes of influenza A viruses tested in this study are denoted by the black dots. (**c**) Application of the pipetting-based immunoassay for enzymatic colour development on the nasal swab samples of grow-finish pigs. (**d**) Application of the pipetting-based immunoassay for enzymatic colour development on the faecal samples of wild birds.

When the pipetting-based immunoassay for enzymatic colour development targeting influenza A virus NP protein was tested with other RNA viruses, colour changes were not only visualised but also detected spectrophotometrically at an absorbance of 1.5–1.9 at 650 nm (Fig. 4a). Other viruses, including bat paramyxovirus B16–40 (BPV)^13^, porcine reproductive and respiratory syndrome virus (PRRSV) strain CP07-401-9^14^, human parainfluenza virus 1 (hPIV1) KBPV-VR-44 strain, dengue virus 3 (DV3) KBVP-VR-30 strain, and dengue virus 4 (DV4) KBPV-VR-31 strain, yielded an absorbance less than 0.6 and were visually distinguishable compared to the swine influenza virus (H3N2).

Seven influenza A virus isolates from humans, dogs, horse, swine, aquatic birds, and chickens (Table 1), which were cultured in embryonated chicken eggs, were also assessed by the pipetting-based immunoassay for enzymatic colour development. Most viral isolates had 256 hemagglutinin (HA) units, while A/California/04/2009 (H1N1) and A/Chicken/Korea/MS96/96 (H9N2) contained 64 and 512 HA units, respectively. As shown in Fig. 4b, the enzymatic colour changes were observed in isolates A/canine/Korea/01/2007 (H3N2), A/California/04/2009 (H1N1), A/Puerto Rico/8/1934 (H1N1), A/aquatic bird/Korea/CN2/2009 (H5N2), and A/Chicken/Korea/MS96/96 (H9N2), except for A/equine/Kyonggi/SA1/2011 (H3N8). In the maximum likelihood phylogenetic tree based on amino acid sequences of the NP protein, the equine influenza viruses (H3N8) constituted their unique clade. The amino acid sequences of the equine influenza virus (H3N8) and A/Puerto Rico/8/1934 (H1N1) displayed 90.3% identity, while others displayed 91.1–94.1% identity (Supplementary Fig. S2).

**Table 1:** Information of various subtype influenza A viruses tested in this study.

Influenza virus strain name	Subtype	HA unit	Host	Genbank No.
A/canine/Korea/01/2007	H3N2	256	Canine	JX163257.1
A/equine/Kyonggi/SA1/2011	H3N8	256	Equine	JX844147.2
A/swine/Korea/P17-4/2017	H3N2	256	Swine	MF624044
A/California/04/2009	H1N1	64	Human	FJ966083.1
A/Puerto Rico/8/1934	H1N1	256	Human	NC_002019.1
A/aquatic bird/Korea/CN2/2009	H5N2	256	Avian	KY584076.1
A/Chicken/Korea/MS96/96	H9N2	512	Avian	AF203787.1

The pipetting-based immunoassay for enzymatic colour development targeting influenza A virus NP protein was assessed using six swine nasal swabs and ten faecal samples from wild birds (Fig. 4c,d). In the case of swine samples, four samples tested positive with influenza A virus M gene-specific reverse transcriptase PCR analysis (WHO, 2011), while all other samples tested negative with one of the commercial lateral flow kits (kit B). The pipetting-based immunoassay for enzymatic colour development could detect two samples among the RT-PCR-positive samples. In case of samples from wild birds, although two samples tested positive upon viral isolation from embryonated chicken eggs, only one of two positive sample tested positive with RT-PCR analysis. The pipetting-based immunoassay for enzymatic colour development used herein notably detected all positive samples upon viral isolation; however, false-positive results were obtained from one sample tested negative with RT-PCR analysis and viral isolation.

### Comparison of results obtained with the lateral flow kit with or without the use of the pipetting-based immunoassay for antigen capture

**Figure 5:**
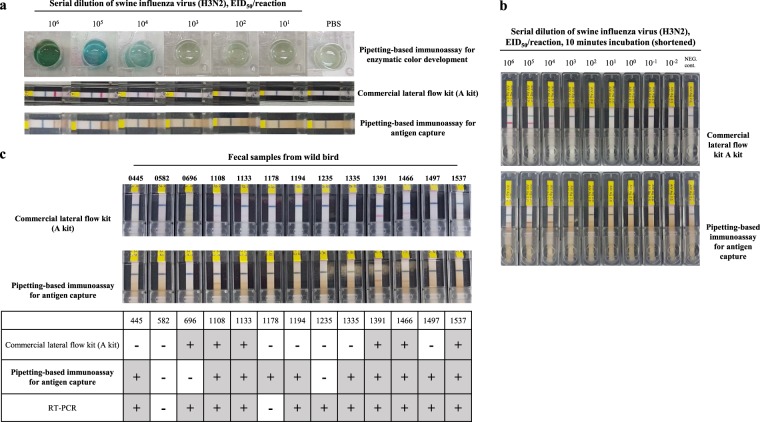
Validation of the pipetting-based immunoassay for antigen capture. (**a**) Comparison of limits of detection among the pipetting-based immunoassay for enzymatic colour development, commercial lateral flow kit, and pipetting-based immunoassay for antigen capture plus commercial lateral flow kit. Ten-fold diluted influenza A virus, A/swine/Korea/P17-4/2017 isolate in PBS was tested. (**b**) Comparison of limits of detection between commercial lateral flow kit alone and pipetting-based immunoassay for antigen capture plus commercial lateral flow kit at the shortened incubation time for the sample reaction (10 min). (**c**) Comparison of detection capability between commercial lateral flow kit alone and pipetting-based immunoassay for antigen capture plus commercial lateral flow kit using faecal samples from wild birds.

The pipetting-based immunoassay for antigen capture was also performed using the commercial lateral flow kit (kit A), which was more sensitive than kit B. When isolate A/swine/Korea/P17-4/2017 was diluted 10-fold with PBS (pH 7.4), the commercial lateral flow kit detected up to 10^4^ EID_50_/reaction when used with the pipetting-based immunoassay for enzymatic colour development (Fig. 5a). However, on performing the pipetting-based immunoassay for antigen capture before the lateral flow assay, the limit of detection improved to 10^2^ EID_50_/reaction. With a reduction in the incubation time from 30 min to 10 min, the limit of detection improved (Fig. 5b). Therefore, these results suggest that application of the pipetting-based immunoassay for antigen capture along with the commercial lateral flow system improved the limit of detection by 100-fold compared to that of the lateral flow system alone.

To determine whether the improved limit of detection was applicable to analysis of clinical samples, 13 faecal samples from wild birds were assessed (Fig. 5c). In RT-PCR analysis, two of the 13 samples tested negative, while the others tested positive. These two samples also tested negative on using the commercial lateral flow kit (kit A), and only six samples tested positive on the lateral flow assay among the 11 positive samples. On performing the pipetting-based immunoassay for antigen capture, the lateral flow kit detected five additional positive samples. However, one sample was false-negative and one was false-positive.

## Discussion

Immunoassays harness specific antigen-antibody interactions to detect target antigens. The specific antigen-antibody complex is usually washed to eliminate unbound material and probed with enzymatic or optic material to quantify it. ELISA is one of the most popular solid phase immunoassays among other different immunoassays. Although ELISA has been used to detect antigens and antibodies, thus serving as the clinical gold standard, conventional ELISA platforms have certain limitations regarding the reaction time and requirement of specific equipment^11,15^. Therefore, ELISA-based POCTs have been developed as an integrated format of the whole ELISA process such as lab-on-compact-disc, moving magnetic nanoparticle-based chip, volumetric bar-chart chip, and single microfluidic chip^11,12,15–17^. Although the integrated form of ELISA is reliable and easy to perform, the complicated fabrication of these devices may serve as a limitation for manufacturers, especially those producing conventional ELISA kits. The pipetting-based immunoassay for enzymatic colour development developed herein is easy to use with a simple set-up for ELISA-based POCTs.

To simplify the washing process for detecting the antigen-antibody complex, we applied detachable ring-type neodymium magnets outside a 1 mL pipette tip (low-binding tip). With this apparatus, the “magnetic bead-capture antibody-targeted protein complex” could be successfully selected by pipetting and was detected with an HRP-conjugated antibody when influenza A virus NP-specific antibodies were used (Fig. 1a,b) and was thus referred to as the pipetting-based immunoassay for enzymatic colour development. For applying this assay to analyse clinical samples, the magnetic pipette tip, buffers, magnetic bead-capture antibody preparation, and HRP-conjugated antibody preparation methods were optimised.

With an increase in the number of recently developed POCTs, accurate and complete data regarding clinical utility, quality, and potential impact of a test on patient-centred clinical outcomes should be considered for the clinical implementation of POCTs^18^. Therefore, we tested the pipetting-based immunoassay for enzymatic colour development with clinical samples such as avian faeces and nasal swabs of pigs. The limit of detection of the assay was similar to that of commercial lateral flow kits, and on analysing clinical samples from pigs and wild birds, the pipetting-based immunoassay for enzymatic colour development detected more samples that tested positive on RT-PCR analysis than a commercial lateral flow kit. However, it could not detect equine influenza A viral isolate A/equine/Kyonggi/SA1/2011(H3N8). This false-negative result may be attributable to the differences in antibody-binding epitopes; however, further follow-up studies are required. The enzymatic colorimetric reaction of pipetting-based ELISA used herein was evaluated through visual inspection, which may be a limitation for quantitative estimation. However, this limitation can be overcome by using portable spectrophotometer such as a smartphone instrument for portable ELISA, which was recently developed^19,20^.

Excluding the enzymatic colour development assay, we attempted to concentrate and purify the target NP of influenza A virus and apply it to the commercial lateral flow system by pipetting-based immunoassay for antigen capture. On assessing the limit of detection using the serially diluted influenza A viral isolate, application of the pipetting-based immunoassay for antigen capture with the commercial lateral flow system improved the limit of detection 100-fold more than that of the lateral flow system alone. When this antigen capture system was used to analyse faecal samples of wild birds, it had a sensitivity of 82%, compared to 55% of the commercial lateral flow kit, considering RT-PCR analysis as the gold standard. Therefore, the pipetting-based immunoassay for antigen capture developed herein can be used for improving the sensitivity of the lateral flow system.

In conclusion, the pipetting-based immunoassay developed herein is a new method facilitating easy washing and purification of the antigen-antibody complex by pipetting, with a potential for application as a clinical POCT. The pipetting-based immunoassay for enzymatic colour development in this study can be evaluated through visual inspection and showed similar limit of detection as the commercial lateral flow kit. However, it may have the limitation of requiring a spectrophotometer to determine cut-off value. The pipetting-based immunoassay for antigen capture could serve as a potential tool to increase limit of detection when combined with commercial lateral flow system.

## Methods

### Materials

A magnetic pipette tip comprising 1 mL pipette tips and ring-like neodymium magnets (ZION, Seoul, Korea) sized 10 mm × 3 mm (R × T) with a hole sized 4.2 mm and 6.5 mm on each side (magnetic ring). Dynabead^TM^ MyOne^TM^ streptavidin T1 (Invitrogen, California, USA) and biotin-conjugated mouse anti-influenza A virus nucleoprotein (NP) monoclonal antibody, clone A3 (Merck, New Jersey, USA) was used for target protein capture. For enzymatic colour development, horseradish peroxidase (HRP)-conjugated mouse anti-influenza A virus NP monoclonal antibody C43 (Abcam, Cambridge, UK) was used (Fig. 1a).

Recombinant influenza A virus NP protein was made to analyse the limit of detection and to establish optimal conditions. Briefly, NP domain (13~459) of influenza A virus (A/Puerto Rico/8-SV11/1934(H1N1), gene accession no. CY105938, NCBI) was expressed using an *Escherichia coli* expression system (Supplementary Fig. S1) and purified by His-tagged affinity chromatography and gel filtration chromatography.

Information regarding the influenza A viruses assessed herein is presented in Table 1. RNA viruses from the following members of other families were selected for the specificity test: BPV^13^, PRRSV strain CP07-401-9^14^, hPIV1 KBPV-VR-44 strain, DV3 KBVP-VR-30 strain, and DV4 KBPV-VR-31 strain. hPIV1, DV3 and DV4 were from Korea Bank for Pathogenic Viruses, Seoul, Korea.

### Generation of the magnetic bead-capture antibody complex and HRP-conjugated antibody

To immobilise biotinylated antibody on streptavidin-coated magnetic beads, 2 µL (2 µg) of biotin-conjugated mouse anti-influenza A virus NP clone A3 and 10 µL (10^8^ beads) of magnetic beads per reaction were mixed in 1.5 mL Eppendorf^®^ tubes and incubated at room temperature for 30 min. Thereafter, the magnetic bead-capture antibody complex was separated from the mixture with two ring-type neodymium magnets and washed four times with 200 µL of 0.1% bovine serum albumin (BSA) in 1X phosphate buffered saline (PBS, pH 7.4). The magnetic bead-capture antibody complex was re-suspended in 12 µL of 1X PBS and used as capture material in this study.

For enzymatic colour development using HRP-conjugated antibody, 10 µL (500 ng) of HRP-conjugated mouse anti-influenza A virus NP monoclonal antibody C43, 10 µL (10^8^ beads) of the magnetic beads and 120 µL of PBS-Tween^®^ 20 (PBS-T) (0.05% Tween^®^ 20 in 1X PBS) were allowed to react at room temperature for 30 min. The mixture was then centrifuged and the supernatant was used for further analysis.

### Pipetting-based immunoassay for enzymatic colour development

The pipetting-based immunoassay for enzymatic colour development was performed using removable magnetic ring-coupled pipette tips in a 4-well plate (SPL Life Sciences, Pocheon, Korea; Fig. 1b). The test samples (recombinant influenza NP protein, influenza A virus isolates, and clinical samples) were diluted with PBS containing 0.1% Triton X-100. Hundred microliters of the prepared sample were then mixed with 12 µL of the prepared magnetic bead-capture antibody complex and 120 µL of the prepared HRP-conjugated antibody and incubated at room temperature for 30 min in 1.5 mL Eppendorf brown tube.

The reactant was gently pipetted 10 times using a removable magnetic ring-coupled pipette tip and the remainder was discarded in the first well of the 4-well plate. The “target antigen (influenza A virus NP)-capture antibody-magnetic bead-HRP-conjugated antibody” bound to the pipette tip by the magnetic ring was washed by pipetting 10 times with 300 µL of PBS-T in second and third wells of the 4-well plate. Finally, after removing the magnetic ring from the pipette tip, the complexes were released in 300 µL of 3,3′,5,5′-tetramethylbenzidine (TMB) solution by pipetting 10 times in the fourth well and incubated at room temperature for 15 min (Supplementary Movie S1). These results were visually confirmed through colour development of the TMB solution, and the absorbance was measured spectrophotometrically at 650 nm.

The optimal magnetic pipette tip apparatus was also determined through comparison between the conventional pipette tip (OHAUS, New Jersey, USA) and low-binding pipette tip (Bioneer, Daejeon, Korea), and between one neodymium and two neodymium magnets. The low-binding pipette tip was expected to reduce non-specific binding of proteins on the interior wall of the tip. Hundred microliters of recombinant NP of influenza A virus (110 µg/mL) in PBS with 0.1% Triton X-100 were used for comparative analysis of the aforementioned method.

### Validation of the pipetting-based immunoassay for enzymatic colour development

Recombinant influenza NP protein (110 µg/mL) was serially diluted 10-fold with PBS. Then, 10^8^ EID_50_/mL of A/swine/Korea/P17-4 isolates was serially 10-fold with 0.1% Triton X-100 in PBS. Hundred microliters of diluted sample were assessed using the pipetting-based immunoassay for enzymatic colour development. The diluted isolates were also assessed using commercial lateral flow kits: Rapid AIV Ag (Bionote, Hwaseong, Korea) and VDRG^®^ AIV Ag Rapid kit 2.0 (Median diagnostics, Chuncheon, Korea).

The 10^8^ EID_50_/ml of A/swine/Korea/P17-4 isolates and allantoic fluid of embryonated chicken egg was serially diluted two-fold with 0.1% Triton X-100 in PBS. The diluted allantoic fluid was used as a negative control. Hundred microliters of diluted samples were used for the pipetting-based immunoassay for enzymatic colour development. For time-based colour development, after the release of the complexes into the TMB solution, absorbance was measured spectrophotometrically at 650 nm for 15 min.

Various subtypes of avian and mammalian of influenza A virus isolates were assessed by the pipetting-based immunoassay for enzymatic colour development. Information regarding these isolates is presented in Table 1. These viruses were diluted 10-fold with 0.1% Triton X-100 and assessed by pipetting-based immunoassay for enzymatic colour development.

To analyse clinical samples, six archived nasal swab samples of grow-finish pigs provided by a field veterinarian were prepared, of which four samples were positive for influenza A virus M gene confirmed by RT-PCR (WHO, 2011), while the others were not. In addition, ten archived faecal samples of wild birds, which were collected from the faeces on the ground of their habitats, were also prepared; among these, two samples were positive for the virus isolate in the embryonated chicken eggs (9–10 d of incubation before viral isolation). All clinical samples were prepared in virus transport medium and assessed by the pipetting-based immunoassay for enzymatic colour development.

### Application of the pipetting-based immunoassay for antigen capture via the lateral flow system

The pipetting-based immunoassay for antigen capture was performed in a manner similar to the pipetting-based immunoassay for enzymatic colour development, with the exception of the HRP-conjugated antibody (Fig. 1c). The test samples (recombinant protein or influenza A virus isolates) were first diluted with PBS containing 0.1% Triton X-100. Two-hundred microliters of the prepared sample was then mixed with 12 µL of the prepared magnetic bead-capture antibody complex and incubated at room temperature for 10 or 30 min. The reactant was gently pipetted 10 times, using a removable magnetic ring-coupled pipette tip and the remainder was discarded into the first well of the 4-well plate. “Target antigen (influenza A virus NP)-capture antibody-magnetic bead” binding to the pipette tip by the magnetic ring were washed by pipetting 10 times with 300 µL of PBS-T in the second and third wells of the 4-well plate. Finally, after removing the magnetic ring from the pipette tip, the complexes were released in 100 µL of PBS by pipetting 10 times in the fourth well. The complexes were directly placed into the lateral flow VDRG^®^ AIV Ag Rapid kit 2.0 (Median diagnostics, Chuncheon, Korea).

### Validation of the pipetting-based immunoassay for antigen capture

A/swine/Korea/P17-4/2017 isolate was diluted 10-fold with PBS (PBS, pH 7.4). First, each dilution was assessed using the VDRG^®^ AIV Ag Rapid kit 2.0 (Median diagnostics, Chuncheon, Korea) in accordance with the manufacturer’s instructions. Briefly, 100 µL of the dilution was mixed with the sample diluent provided in the kit and inoculated into the lateral flow kit. Second, the dilution was assessed by the pipetting-based immunoassay for antigen capture by the lateral flow system.

To compare the clinical samples, 13 faecal samples from wild birds were diluted in PBS and assessed by the pipetting-based immunoassay for antigen capture and commercial lateral flow kit. RNA was also extracted from the samples and RT-PCR (WHO, 2011), which is widely considered the gold standard, was performed.

## Supplementary information

supplementary dataset

Movie S1

## References

[1] Sharma S, Zapatero-Rodríguez J, Estrela P, O’Kennedy R (2015). Point-of-care diagnostics in low resource settings: present status and future role of microfluidics. Biosensors.

[2] Hsieh H, Dantzler J, Weigl B (2015). Analytical tools to improve optimization procedures for lateral flow assays. Diagnostics.

[3] Koczula KM, Gallotta A (2016). Lateral flow assays. Essays Biochem..

[4] Tang D (2009). Magnetic nanogold microspheres-based lateral-flow immunodipstick for rapid detection of aflatoxin B2 in food. Biosens. Bioelectron..

[5] Zou Z (2010). Quantum dot-based immunochromatographic fluorescent biosensor for biomonitoring trichloropyridinol, a biomarker of exposure to chlorpyrifos. Anal. Chem..

[6] Peruski AH, Peruski LF (2003). Immunological methods for detection and identification of infectious disease and biological warfare agents. Clin. Diagn. Lab. Immunol..

[7] Sin ML, Mach KE, Wong PK, Liao JC (2014). Advances and challenges in biosensor-based diagnosis of infectious diseases. Expert. Rev. Mol. Diagn..

[8] Lequin RM (2005). Enzyme immunoassay (EIA)/enzyme-linked immunosorbent assay (ELISA). Clin. Chem..

[9] Souf S (2016). Recent advances in diagnostic testing for viral infections. Biosci. Horiz..

[10] Mark D, Haeberle S, Roth G, von Stetten F, Zengerle R (2010). Microfluidic lab-on-a-chip platforms: requirements, characteristics and applications. Chem. Soc. Rev..

[11] Song Y (2012). Multiplexed volumetric bar-chart chip for point-of-care diagnostics. Nat. Commun..

[12] Song, Y., Li, Y. & Qin, L. Volumetric Bar-Chart Chips for Biosensing in *Biomedical Nanotechnology*. 105–115 (Humana Press, New York, 2017). 10.1007/978-1-4939-6840-4_7 28238132

[13] Noh, J. Y. *et al*. Isolation and characterization of novel bat paramyxovirus B16-40 potentially belonging to the proposed genus Shaanvirus. *Sci. Rep*. **8**, 12533–12533 (2018). 10.1038/s41598-018-30319-7 PMC6105681 30135435

[14] Kim HK (2009). Genetic analysis of ORF5 of recent Korean porcine reproductive and respiratory syndrome viruses (PRRSVs) in viremic sera collected from MLV-vaccinating or non-vaccinating farms. J. Vet. Sci..

[15] Thiha A, Ibrahim F (2015). A colorimetric enzyme-linked immunosorbent assay (ELISA) detection platform for a point-of-care dengue detection system on a lab-on-compact-disc. Sensors.

[16] Adel Ahmed H, Azzazy HME (2013). Power-free chip enzyme immunoassay for detection of prostate specific antigen (PSA) in serum. Biosens. Bioelectron..

[17] Liu D (2017). A fully integrated distance readout ELISA-Chip for point-of-care testing with sample-in-answer-out capability. Biosens. Bioelectron..

[18] Drain PK (2014). Diagnostic point-of-care tests in resource-limited settings. Lancet Infect. Dis..

[19] Long KD, Yu H, Cunningham BT (2014). Smartphone instrument for portable enzyme-linked immunosorbent assays. Biomed. Opt. Express..

[20] Berg B (2015). Cellphone-based hand-held microplate reader for point-of-care testing of enzyme-linked immunosorbent assays. ACS Nano..

[21] Tamura K, Stecher G, Peterson D, Filipski A, Kumar S (2013). MEGA6: molecular evolutionary genetics analysis version 6.0. Mol. Biol. Evol..

