# Supplementary material for: Pipetting-based immunoassay for point-of-care testing: Application for detection of the influenza A virus
Source: Sci Rep. 2019 Nov 13;9:16661. doi: 10.1038/s41598-019-53083-8 (PMC6853919; doi:10.1038/s41598-019-53083-8)
Supplement: Supplementary file 1 — supplementary dataset [file 41598_2019_53083_MOESM1_ESM.pdf]

Supplementary Materials for

Pipetting-based immunoassay for point-of-care testing: Application for detection of the influenza A virus

Ji Yeong Noh[1,2, †], Sun-Woo Yoon[2,3, †], Youngji Kim[2], Thi Van Lo[2,3], Min-Ju Ahn[2,3], Min-Chul Jung[2,3], Tran Bac Le[2,3], Woonsung Na[4], Daesub Song[4], Van Phan Le[5], Seungjoo Haam[6], Dae-Gwin Jeong[2,3,*], and Hye Kwon Kim[1,2,*]

***Address for Corresponding authors:**

Dae Gwin Jeong, MSc, PhD (e-mail: dgjeong@kribb.re.kr; tel: +82-42-879-8411)

Hye Kwon Kim, DVM, PhD (e-mail: khk1329@chungbuk.ac.kr ; tel: +82-43-261-2302)

The PDF file include

Fig. S1. Expression and Purification of recombinant influenza A virus nucleocapsid protein

Fig. S2. Sequence identity matrix of nucleocapsid protein amino acids of influenza A viruses in this study

Other supplement material for this manuscript includes the following:

Movie. S1. Pipetting-based immunoassay for enzymatic color development

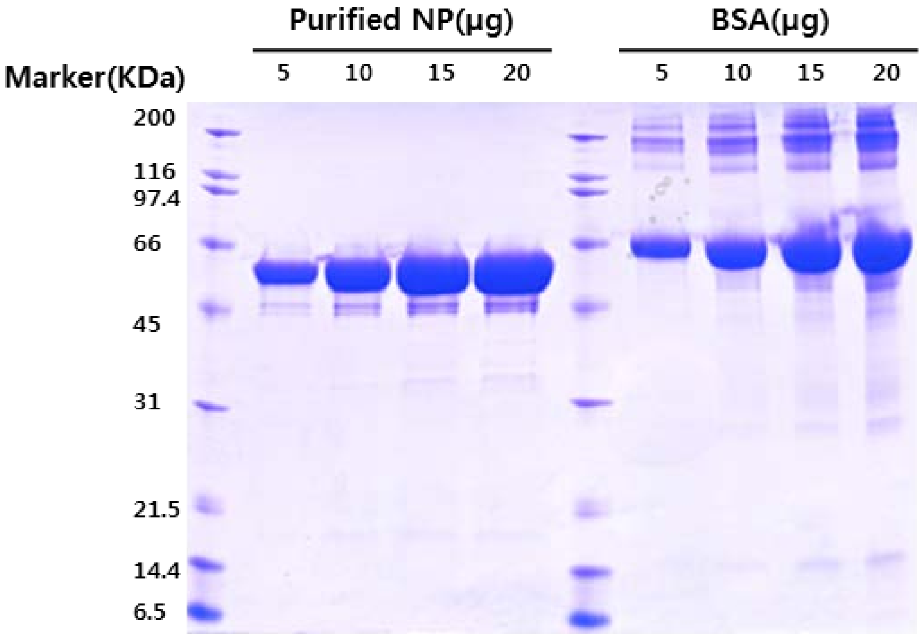

Fig. S1. Expression and Purification of recombinant influenza A virus nucleocapsid protein

The nucleocapsid protein (NP) domain (13~459) of influenza A virus (A/Puerto Rico/8-SV11/1934(H1N1), gene accession No. CY105938, NCBI) was amplified via PCR from cDNA, using the following primers: H1N1NP- NdeI forward (5'-GGAATTCATATGGAGACTGATGGAG AACGCCAG-3') and H1N1NP-EcoRI reverse (5'-CCGGAATTCTTACTCCTCTGCATTG TCTCCGAAG-3'). The PCR conditions are as follows; i) Denaturation for 5 min at 94°C, ii) Secondary denaturation for 1 min at 94 °C, iii) Annealing for 1 min at 56°C, iv) Polymerization for 1 min and 30 sec at 72 °C, v) Repetition of steps ii) – iv) for 30 cycles, vi) Reaction for 10 min at 72 °C and the end of reaction. The PCR products were ligated at the NdeI and EcoRI restriction sites of pET28a (Novagen, USA) containing a hexahistidine tag, Maltose binding protein (MBP), and a TEV protease recognition site at the N-terminus of the protein. NP was expressed in BL21 DE3 Codon plus E. coli cells (Invitrogen). The pET28a-6His-MBP-TEV-NP vector-containing E. coli cells were incubated with 0.2 mM IPTG (final concentration) to induce protein expression. After 16~18 h of incubation at 18°C, the cells were harvested via centrifugation at 6,000 X g for 10min. The supernatant was eliminated, and the cells were suspended in 30 ml of lysis/binding buffer (50 mM Tris-HCl (pH 7.5), 500 mM NaCl, 1 mM phenylmethylsulfonyl fluoride, 4 mM 2-mercaptoethanol, and 10% glycerol) per liter culture volume and sonicated thrice on ice for 30 s at 60% power. The lysate was cleared via centrifugation at 28,000 X g for 50min. The protein was purified through Ni-NTA Superflow (Qiagen, Germany) metal affinity chromatography. The eluate was incubated with TEV protease at 18°C overnight to eliminate the MBP tag and then passed through a Heparin High Performance (HP) column (GE Healthcare USA). NP was eluted in a 0 to 1.5 M NaCl gradient and further purified via Superdex 200 size exclusion chromatography (GE Healthcare, USA). The protein was isolated via a final purification step in 2X PBS and its concentration and purity were determined via the Bradford assay and SDS-PAGE (Bio-Rad, USA), using standard BSA as a control.

Fig. S2. Sequence identity matrix of nucleocapsid protein amino acids of influenza A viruses in this study

Seq->	*JX163257	*JX844147	*FJ966083.	*NC_00201	*AF203787	*MF62404	*KY584076	DQ124158	KM359837	KC599552.	DQ124184	AY291288.	KC986393.	AY790308.	CY107036.	KC951191.	AY129159.	KC951186	JF820286.1	KC508568.	KT221067.	AB497023.	AY342427.	KJ174938.1	KM455873	AB539740.	KC782314.	MG83111	MG830865
*JX163257.1 A/canine/Korea/01/2007(H3N2)	ID	0.931	0.929	0.923	0.963	0.929	0.975	0.935	0.931	0.985	0.935	0.941	0.937	0.893	0.925	0.927	0.919	0.929	0.939	0.929	0.973	0.959	0.977	0.955	0.961	0.929	0.927	0.909	0.929
*JX844147.2 A/equine/Kyonggi/SA1/2011(H3N8)	0.931	ID	0.911	0.903	0.931	0.907	0.935	0.991	0.983	0.927	0.991	0.975	0.981	0.861	0.907	0.907	0.899	0.909	0.913	0.907	0.937	0.919	0.941	0.915	0.921	0.911	0.909	0.901	0.905
*FJ966083.1 A/California/04/2009(H1N1)	0.929	0.911	ID	0.915	0.933	0.979	0.941	0.913	0.909	0.927	0.913	0.917	0.917	0.869	0.973	0.985	0.969	0.991	0.919	0.977	0.939	0.933	0.943	0.921	0.931	0.997	0.995	0.897	0.991
*NC_002019.1 A/Puerto Rico/8/1934(H1N1)	0.923	0.903	0.915	ID	0.935	0.911	0.941	0.907	0.909	0.917	0.907	0.907	0.911	0.869	0.909	0.909	0.903	0.911	0.927	0.915	0.939	0.931	0.943	0.919	0.927	0.913	0.911	0.917	0.911
*AF203787.1 A/Chicken/Korea/MS96/96(H9N2)	0.963	0.931	0.933	0.935	ID	0.931	0.983	0.935	0.935	0.953	0.935	0.949	0.937	0.905	0.929	0.929	0.923	0.931	0.951	0.931	0.981	0.969	0.985	0.961	0.969	0.933	0.931	0.905	0.931
*MF624044 A/swine/Korea/P17-4/2017(H3N2)	0.929	0.907	0.979	0.911	0.931	ID	0.939	0.909	0.905	0.927	0.909	0.913	0.913	0.865	0.973	0.969	0.973	0.975	0.917	0.989	0.937	0.935	0.941	0.923	0.929	0.977	0.975	0.893	0.971
*KY584076.1 A/aquatic bird/Korea/CN2/2009(H5N2)	0.975	0.935	0.941	0.941	0.983	0.939	ID	0.939	0.939	0.965	0.939	0.949	0.941	0.911	0.935	0.937	0.927	0.939	0.959	0.939	0.989	0.977	0.993	0.969	0.977	0.941	0.939	0.913	0.941
DQ124158.1 A/canine/Florida/242/2003(H3N8)	0.935	0.991	0.913	0.907	0.935	0.909	0.939	ID	0.991	0.929	1	0.983	0.987	0.865	0.909	0.909	0.899	0.911	0.915	0.909	0.941	0.921	0.945	0.917	0.923	0.913	0.911	0.905	0.907
KM359837.1 A/canine/VT/11039/2013(H3N8)	0.931	0.983	0.909	0.909	0.935	0.905	0.939	0.991	ID	0.925	0.991	0.975	0.979	0.861	0.905	0.905	0.895	0.907	0.917	0.905	0.941	0.921	0.941	0.915	0.921	0.909	0.907	0.903	0.907
KC599552.1 A/canine/Thailand/CU-DC5299/2012(H3N2)	0.985	0.927	0.927	0.917	0.953	0.927	0.965	0.929	0.925	ID	0.929	0.931	0.931	0.883	0.919	0.927	0.917	0.925	0.939	0.931	0.963	0.961	0.967	0.953	0.955	0.927	0.925	0.901	0.927
DQ124184.1 A/equine/Ohio/1/2003(H3N8)	0.935	0.991	0.913	0.907	0.935	0.909	0.939	1	0.991	0.929	ID	0.983	0.987	0.865	0.909	0.909	0.899	0.911	0.915	0.909	0.941	0.921	0.945	0.917	0.923	0.913	0.911	0.905	0.907
AY291288.1 A/equine/Kentucky/1/81(H3N8)	0.941	0.975	0.917	0.907	0.949	0.913	0.949	0.983	0.975	0.931	0.983	ID	0.975	0.875	0.913	0.913	0.907	0.915	0.921	0.913	0.947	0.931	0.955	0.925	0.933	0.917	0.915	0.897	0.911
KC986393.2 A/equine/Heilongjiang/SS1/2013(H3N8)	0.937	0.981	0.917	0.911	0.937	0.913	0.941	0.987	0.979	0.931	0.987	0.975	ID	0.869	0.909	0.913	0.903	0.915	0.917	0.913	0.943	0.923	0.947	0.919	0.925	0.917	0.915	0.907	0.911
AY790308.1 A/swine/Korea/S452/2004(H9N2)	0.893	0.861	0.869	0.869	0.905	0.865	0.911	0.865	0.861	0.883	0.865	0.875	0.869	ID	0.869	0.867	0.855	0.867	0.877	0.865	0.907	0.899	0.915	0.891	0.901	0.869	0.867	0.847	0.867
CY107036.1 A/swine/North Carolina/A01076199/2010(H3N2)	0.925	0.907	0.973	0.909	0.929	0.973	0.935	0.909	0.905	0.919	0.909	0.913	0.909	0.869	ID	0.963	0.967	0.969	0.907	0.975	0.931	0.929	0.935	0.923	0.931	0.971	0.969	0.891	0.965
KC951191.1 A/swine/Minnesota/A01327763/2012(H3N2)	0.927	0.907	0.985	0.909	0.929	0.969	0.937	0.909	0.905	0.927	0.909	0.913	0.913	0.867	0.963	ID	0.959	0.993	0.915	0.971	0.935	0.935	0.939	0.923	0.929	0.987	0.985	0.895	0.981
AY129159.1 A/Swine/Korea/CY02/02(H1N2)	0.919	0.899	0.969	0.903	0.923	0.973	0.927	0.899	0.895	0.917	0.899	0.907	0.903	0.855	0.967	0.959	ID	0.965	0.907	0.975	0.925	0.923	0.929	0.913	0.917	0.967	0.965	0.881	0.961
KC951186.1 A/swine/Ohio/A01116889/2011(H1N2)	0.929	0.909	0.991	0.911	0.931	0.975	0.939	0.911	0.907	0.925	0.911	0.915	0.915	0.867	0.969	0.993	0.965	ID	0.917	0.973	0.937	0.933	0.941	0.921	0.931	0.993	0.991	0.897	0.987
JF820286.1 A/swine/Jiangsu/s16/2011(H1N1)	0.939	0.913	0.919	0.927	0.951	0.917	0.959	0.915	0.917	0.939	0.915	0.921	0.917	0.877	0.907	0.915	0.907	0.917	ID	0.917	0.953	0.947	0.961	0.935	0.943	0.919	0.917	0.899	0.917
KC508568.1 A/swine/Indiana/A01260029/2012(H1N1)	0.929	0.907	0.977	0.915	0.931	0.989	0.939	0.909	0.905	0.931	0.909	0.913	0.913	0.865	0.975	0.971	0.975	0.973	0.917	ID	0.937	0.939	0.941	0.929	0.929	0.975	0.973	0.893	0.969
KT221067.1 A/goose/Jiangsu/QD5/2014(H5N8)	0.973	0.937	0.939	0.939	0.981	0.937	0.989	0.941	0.941	0.963	0.941	0.947	0.943	0.907	0.931	0.935	0.925	0.937	0.953	0.937	ID	0.971	0.987	0.963	0.971	0.939	0.937	0.915	0.939
AB497023.1 A/duck/Egypt/D1Li5/2007(H5N1)	0.959	0.919	0.933	0.931	0.969	0.935	0.977	0.921	0.921	0.961	0.921	0.931	0.923	0.899	0.929	0.935	0.923	0.933	0.947	0.939	0.971	ID	0.975	0.973	0.979	0.933	0.931	0.903	0.933
AY342427.1 A/chicken/Netherlands/1/03(H7N7)	0.977	0.941	0.943	0.943	0.985	0.941	0.993	0.945	0.941	0.967	0.945	0.955	0.947	0.915	0.935	0.939	0.929	0.941	0.961	0.941	0.987	0.975	ID	0.969	0.977	0.943	0.941	0.919	0.941
KJ174938.1 A/chicken/Henan/Q7/2013(H5N2)	0.955	0.915	0.921	0.919	0.961	0.923	0.969	0.917	0.915	0.953	0.917	0.925	0.919	0.891	0.923	0.923	0.913	0.921	0.935	0.929	0.963	0.973	0.969	ID	0.977	0.921	0.919	0.905	0.921
KM455873.1 A/Lengshuitan/11197/2013(H9N2)	0.961	0.921	0.931	0.927	0.969	0.929	0.977	0.923	0.921	0.955	0.923	0.933	0.925	0.901	0.931	0.929	0.917	0.931	0.943	0.929	0.971	0.979	0.977	0.977	ID	0.931	0.929	0.911	0.931
AB539740.1 A/Shanghai/P1/2009(H1N1)	0.929	0.911	0.997	0.913	0.933	0.977	0.941	0.913	0.909	0.927	0.913	0.917	0.917	0.869	0.971	0.987	0.967	0.993	0.919	0.975	0.939	0.933	0.943	0.921	0.931	ID	0.997	0.895	0.993
KC782314.1 A/Minnesota/16/2009(H1N1)	0.927	0.909	0.995	0.911	0.931	0.975	0.939	0.911	0.907	0.925	0.911	0.915	0.915	0.867	0.969	0.985	0.965	0.991	0.917	0.973	0.937	0.931	0.941	0.919	0.929	0.997	ID	0.893	0.991
MG831119.1 A/Virginia/37/2017(H3N2)	0.909	0.901	0.897	0.917	0.905	0.893	0.913	0.905	0.903	0.901	0.905	0.897	0.907	0.847	0.891	0.895	0.881	0.897	0.899	0.893	0.915	0.903	0.919	0.905	0.911	0.895	0.893	ID	0.893
MG830865.1 A/West Virginia/31/2017(H1N1)	0.929	0.905	0.991	0.911	0.931	0.971	0.941	0.907	0.907	0.927	0.907	0.911	0.911	0.867	0.965	0.981	0.961	0.987	0.917	0.969	0.939	0.933	0.941	0.921	0.931	0.993	0.991	0.893	ID
